# Visualising Androgen Receptor Activity in Male and Female Mice

**DOI:** 10.1371/journal.pone.0071694

**Published:** 2013-08-07

**Authors:** D. Alwyn Dart, Jonathan Waxman, Eric O. Aboagye, Charlotte L. Bevan

**Affiliations:** Department of Surgery & Cancer, Imperial Centre for Translational and Experimental Medicine, Imperial College London, London, United Kingdom; Montana State University, United States of America

## Abstract

Androgens, required for normal development and fertility of males and females, have vital roles in the reproductive tract, brain, cardiovascular system, smooth muscle and bone. Androgens function via the androgen receptor (AR), a ligand-dependent transcription factor. To assay and localise AR activity in vivo we generated the transgenic “ARE-Luc” mouse, expressing a luciferase reporter gene under the control of activated endogenous AR. *In vivo* imaging of androgen-mediated luciferase activity revealed several strongly expressing tissues in the male mouse as expected and also in certain female tissues. In males the testes, prostate, seminal vesicles and bone marrow all showed high AR activity. In females, strong activity was seen in the ovaries, uterus, omentum tissue and mammary glands. In both sexes AR expression and activity was also found in salivary glands, the eye (and associated glands), adipose tissue, spleen and, notably, regions of the brain. Luciferase protein expression was found in the same cell layers as androgen receptor expression. Additionally, mouse AR expression and activity correlated well with AR expression in human tissues. The anti-androgen bicalutamide reduced luciferase signal in all tissues. Our model demonstrates that androgens can act in these tissues directly via AR, rather than exclusively via androgen aromatisation to estrogens and activation of the estrogen receptor. Additionally, it visually demonstrates the fundamental importance of AR signalling outside the normal role in the reproductive organs. This model represents an important tool for physiological and developmental analysis of androgen signalling, and for characterization of known and novel androgenic or antiandrogenic compounds.

## Introduction

Androgens are responsible for masculinization of male body tissues, e.g. development of the internal and external genitalia, and in both sexes, the androgen surge at puberty drives development of secondary sexual characteristics, e.g. increased skeletal muscle bulk, voice deepening, and axillary and pubic hair growth [1]. The principal circulating androgen is testosterone, most of which is synthesised from androgenic precursors in the gonads. Less potent androgens (such as dehydroepiandrosterone) are also produced by the adrenal glands, and some peripheral conversion of adrenal androgens to testosterone also occurs (more significantly in females). Androgens act via the androgen receptor (AR), a ligand-activated transcription factor which has structural homology to the glucocorticoid receptor (GR), progesterone receptor (PR) and mineralocorticoid receptor (MR), and to a lesser extent the estrogen receptor (ERα).

Normal male physiological development requires a controlled pattern of gene expression from fertilisation to gestation, and involves expression of the SRY transcription factor (Sex-determining Region Y) from the Y chromosome to initiate phenotypic male sexual differentiation and testis development [2–4]. These early testes secrete testosterone, which drives differentiation and growth of the genital tissues and Wolffian structures, while metabolism to the more potent agonist dihydrotestosterone (DHT) by 5alpha-reductase enzymes in target cells drives growth of the prostate and phallus. Reduced androgen signalling in males can result in undervirilization and infertility, while increased androgen signalling is associated with increased prostate cancer risk [5,6]. In the male mouse brain, the sexually dimorphic regions express high levels of AR and male development is believed to result from exposure to testicular androgens – indeed it can be mimicked in females by prenatal androgen exposure [7,8].

In the female, androgens are produced by the adrenal, ovarian and adipose tissues but circulate at a lower level than in the male. A key function of androgens in females is aromatisation to estrogens, but the AR is expressed in several female tissues, e.g. mammary gland, uterus, vulvar epithelium, vaginal mucosa and in ovarian follicles where it maintains follicle health during ovulation [9–12]. In the female brain, androgens regulate initiation of sexual activity, libido and mating behaviour [13,14]. Testosterone importance in the female is demonstrated by profound effects of testosterone insufficiency in menopausal or post-oophorectomied females, including diminished libido, fatigue, hair loss, osteopenia, osteoporosis and decreased body mass [15,16].

AR function and localization may be assessed by immunohistochemistry or binding of radioactive ligands [17], but such studies involve tissue analysis and the sacrifice of many animals, and cannot determine the final level of AR activity since each tissue expresses a repertoire of transcription factors and coactivators that act along with the AR, resulting in tissue-specific target gene expression. We have developed an improved transgenic model for AR activity utilising firefly luciferase as a reporter gene, allowing *in vivo* imaging in live anaesthetized animals. Luciferase (*Luc2P*), being non-mammalian, gives very low background signal and has been engineered to contain degradation signals (hPEST) for rapid protein turnover, allowing real-time signal responses to changes in gene activation. Furthermore, consecutive longitudinal images can be taken using the same animal.

The consensus response element for the AR is identical to that of the closely related GR, PR and MR - a hexameric bipartite binding site, comprising of an inverted repeat of TGTTCT with a three nucleotide spacer - and therefore could not be used due to lack of discrimination [18]. However, promoter/enhancer analysis of androgen-responsive genes has revealed additional non-consensus response elements with apparent AR specificity. Two well-studied examples are the rat dorsal prostate-specific probasin (PB) and the prostate trans-epithelial transporter of IgM (secretory component or *SC*1.2) genes [19–22]. These specific AREs differ from the inverted repeat, having greater similarity to a direct repeat of the same sequence but with a change of T-G in the first base - increasing AR specificity while excluding cross-talk with other steroid receptors [23,24]. Our reporter was constructed by cloning the *SC*1.2-ARE upstream of a minimal thymidine kinase promoter, containing a TATA box for RNA polymerase binding. We excluded adjacent endogenous DNA sequences in order to rule out binding of other transcription factors that could render the response tissue-specific, as seen for prostate specific antigen (PSA) gene promoter-reporter fusions [25]. Additionally, targeted genome integration of our reporter gene eliminated deleterious effects of random integration such as heterochromatin-induced gene silencing. Further, the reporter is activated by endogenous AR protein, thus will recapitulate the endogenous androgen responses.

## Results

### Generation of a highly specific androgen reporter luciferase construct

**Figure 1:**
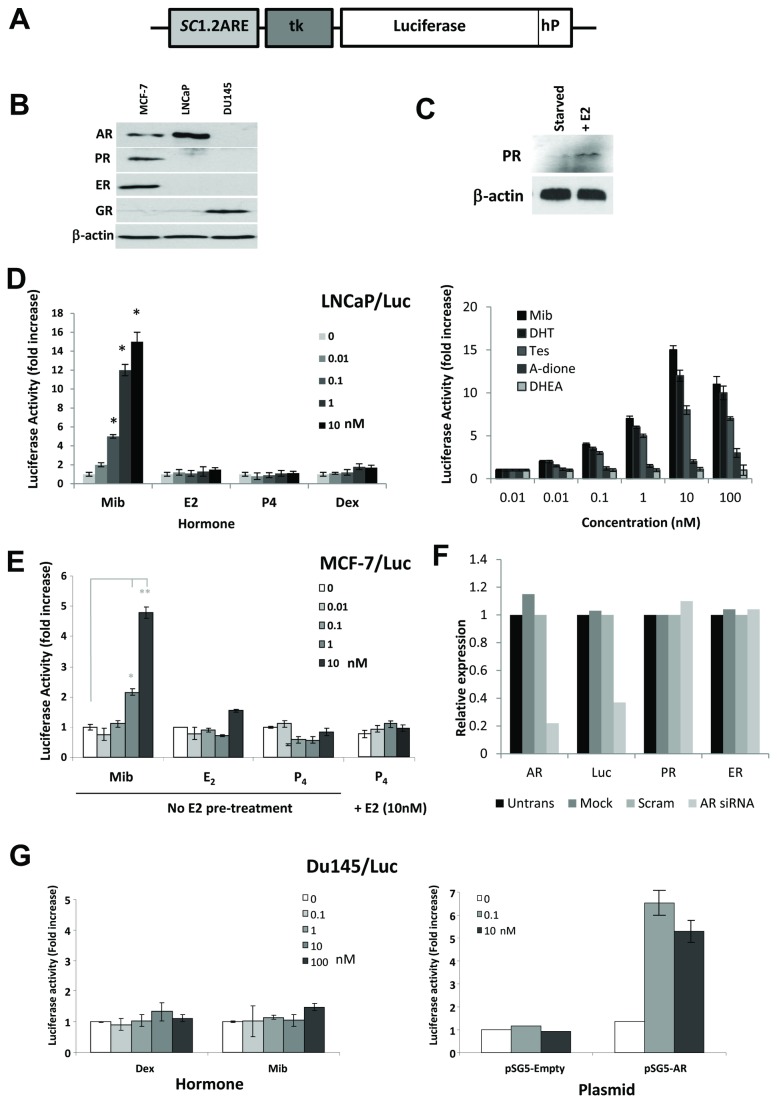
Analysis of the androgen receptor reporter construct in stably transfected cells. (**A**) Schematic representation of the ARE-tk-Luc androgen receptor reporter construct (hP = hPEST degradation signal). (**B**) Western blot analysis of steroid receptor expression in MCF-7, LNCaP and Du145 cells growing in full media. (**C**) Western blot analysis of PR expression in hormone starved MCF-7 cells -/+ E2 treatment for 24hr. (**D**), Luciferase activity from hormone treated LNCaP/Luc cells – treated for 24hours with 0-10nM mibolerone (Mib), estrogen (E2), progesterone (P4) and dexamethasone (Dex) (left hand side) and with 0-100nM of the androgens - (Mib), dihydrotestosterone (DHT), testosterone (Tes), androstenedione (A-dione) and dehydroepiandrosterone (DHEA) for 24 hours (right hand side). (**E**) Luciferase activity from hormone treated MCF-7/Luc cells – treated for 24hours with 0-10nM Mib, E2 or P4 or with additional 24hr pre-treatment with E2 (10nM) to induce PR expression. (**F**) Q-PCR quantification of relative expression of the steroid receptors AR, ER, and PR and luciferase transcripts in MCF-7/Luc cells, grown in full medium, transfected with siRNA against androgen receptor. (**F**) Luciferase activity from Du145/Luc cells treated with 0-100nM dex or mib (left hand side) or pre-transfected with an additional AR or empty vector expression constructs (right hand side). **P<0.01, *P<0.05 (t-test analysis).

Our aim was to create a reporter construct that responds specifically to active AR, with minimal crosstalk with other steroids/steroid receptors. Activity was tested initially by transient transfection into steroid receptor negative COS-1 cells alongside vectors expressing human AR, GR, ERα, and PR, and also in cells expressing one or two endogenous receptors. The *SC*1.2-ARE was used as, of several tested, it showed the greatest androgen induction and specificity for AR without activation by other transfected or endogenous steroid receptors, within the physiological range of steroid hormones (see Dart et al., 2009 supplemental data) [26]. The SC1.2-ARE sequence was cloned upstream of a minimal thymidine kinase (tk) promoter to drive luciferase (*luc2P*) gene expression (Figure **1A**). Subsequently the construct was stably integrated into cell lines known to endogenously express steroid receptors (Figure **1B & C**) - LNCaP cells (AR^+^), MCF-7 cells (ERα^+^/PR^+^/AR^+^) and Du145 cells (AR^-^/GR^+^), and treated with the cognate ligands. LNCaP/Luc cells showed good induction of the reporter when treated with mibolerone, and dose-dependent response to a broad range of different androgens, with no significant response to estrogen (E_2_), progesterone (P_4_) or dexamethasone (Dex) (Figure **1D**). MCF-7/Luc cells showed good androgen-induced luciferase responsiveness but were devoid of any significant luciferase responses to E_2_ or P_4_ (Figure **1E**). In the case of P_4_, this was seen even when cells were pretreated with E_2_ to induce PR expression (Figure **1C & E**). Also in MCF-7/Luc cells, siRNA against the AR resulted in a reduced level of AR transcripts as expected and concurrently reduced the luciferase expression in the presence of androgen (Figure **1F**). No luciferase response was seen in dex-treated Du145/Luc cells (Figure **1G** **, left hand side**), but upon transfection with an AR-expressing vector, an androgen-induced response was seen, confirming the reporter was functional (Figure **1G** **, right hand side**). In summary, this luciferase reporter responds specifically to AR activation in a range of cell lines.

### Luciferase expression in transgenic mice

**Figure 2:**
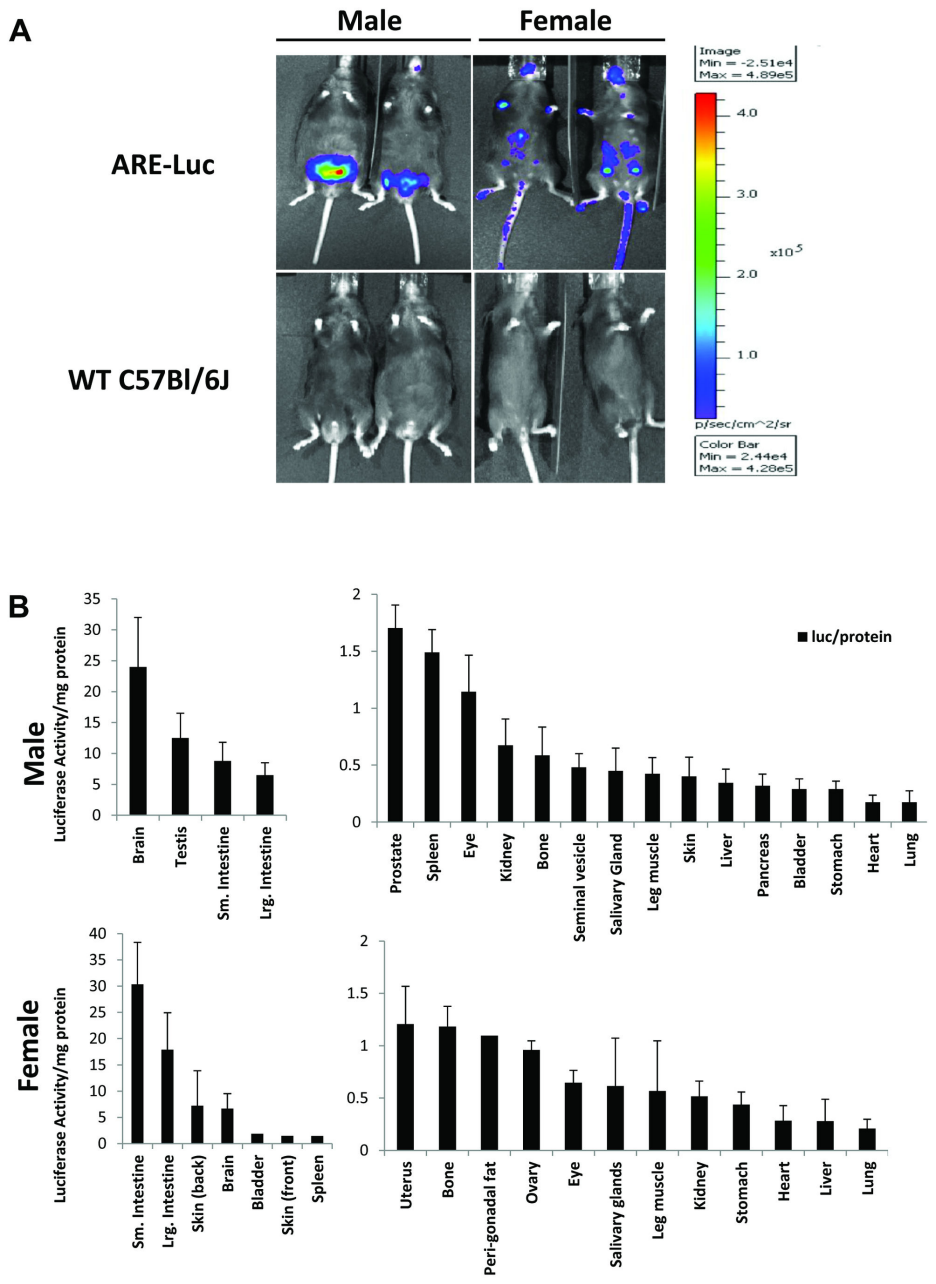
Androgen receptor reporter activity in transgenic mice. (**A**), Bioluminescent imaging of male and female ARE-Luc and C57/Bl/6J wild type mice, injected with 150mg/kg luciferin substrate, and imaged with a CCCD camera after 10 minutes. Figure represents a greyscale photograph overlaid with a pseudocolour representation of bioluminescence; scale represents photons/sec/cm^2^. (**B**), Luciferase enzymatic assays from tissue homogenates taken from ARE-Luc mice, normalised to protein content (Bradford assay). Male mice upper panel, female mice lower panel.

The S.C. 1.2 ARE luciferase vector was used to generate transgenic ARE-Luc mice (details in methods and supplemental data Figure S1). First, we investigated AR transcriptional activity as measured by luciferase activity in adult male and female mice of approximately 6-10 weeks of age. ARE-Luc mice, along with wild type controls, were injected with luciferin substrate (150 mg/kg subcutaneous (*s.c.*)) and imaged to detect bioluminescence. Male ARE-Luc mice showed significant bioluminescence/photon emission observed in body areas corresponding to the reproductive organs (Figure **2A**). Minimal light emission was seen from the skin and limbs of the male mice. Female ARE-Luc mice showed distinct differences to the males, with bioluminescence from regions corresponding to the mammary glands, multiple abdominal signals and regions of skin. Overall total body bioluminescence was approx. three times stronger in males than females. Wild type mice showed no bioluminescence as expected (Figure **2A** **, lower panel**).

Intra peritoneal (*i.p.*) injection showed faster kinetics of luciferin light emission, reaching steady state maximum around 5 minutes, compared to 10 minutes for the *s.c.* route (Figure **S2**). However, the *s.c.* route was utilised to avoid signal bias towards gonadal tissue adjacent to the *i.p.* site. The luciferase signal remained stable for over 20 minutes post injection.

### Androgen receptor activity in specific mouse tissues

Luciferase activity (normalised to protein content) was measured from lysates of several different tissues from male and female ARE-Luc mice aged 6-10 weeks. In male mice, the tissues exhibiting highest luciferase activity/mg protein content were the brain and the testes. The intestines, prostate, spleen, and eye showed a modest luciferase activity and of the tissues measured the lowest activity in males was seen in the stomach, heart and lung (Figure **2B** **, upper panel**).

In female mice, the strongest luciferase activity/mg protein signal was seen in the intestines (omentum included), skin, brain, bladder and spleen. Modest luciferase activity was seen in the uterus, bone, gonadal fat pads, and ovaries (Figure **2B** **, lower panel**). Again, the lowest activity was seen in the heart and lung, as well as liver.

**Figure 3:**
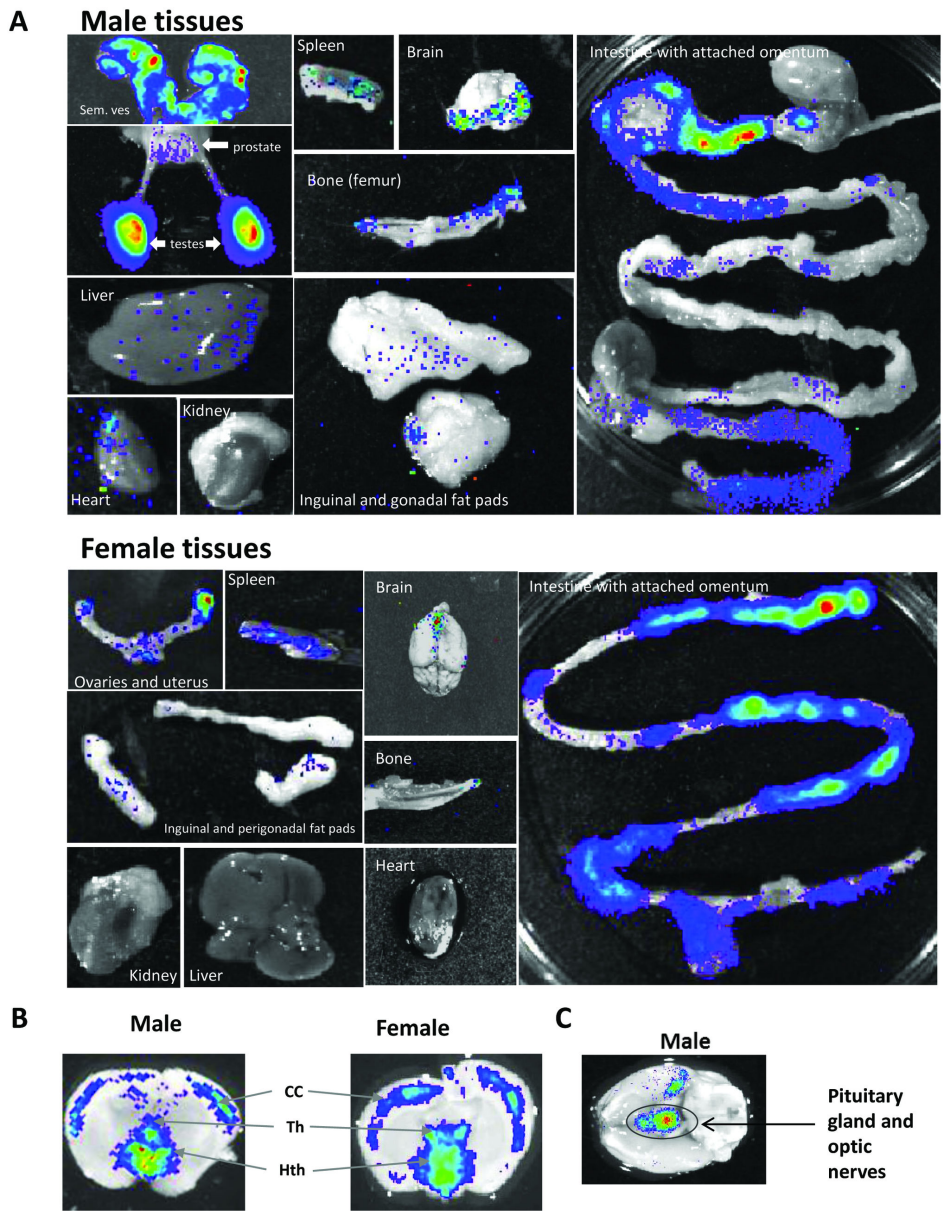
Bioluminescence analysis of *ex vivo* tissues. *Ex vivo* bioluminescence imaging of tissues taken from ARE-Luc mice injected with 150mg/kg luciferin substrate and killed and dissected after 10 minutes. (**A**), Images of male tissues (upper panel) and female mice (lower panel). (**B**), Images of coronal sections through the cerebral cortex of a male and female mouse brain. CC - Cerebral Cortex, Th - Thalamus, Hth - Hypothalamus. (**c**), Image of the underside of the male brain, showing pituitary and optic nerve bundles. Figure represents a greyscale photograph overlaid with a pseudocolour representation of bioluminescence. Images are representative of the pattern seen in several mice.

Tissues were dissected out and imaged *ex vivo* 10 minutes after luciferin injection. In male ARE-Luc mice, the testes, prostate and the seminal vesicles accounted for the majority of the light emitted from live male mice (Figure **3A** **, upper panel**) – the brain signal being masked by the skull until excised. Moderate luciferase activity was also seen in the spleen, areas of the brain, bone/bone marrow and omentum attached to the intestine. Weaker but detectable AR-mediated luciferase activity was also seen in the liver, perigonadal fat deposits and specific regions of the heart (atrial region/vascular bundle).

In female mice, *ex vivo* imaging of tissues revealed strong luciferase signal in the ovaries and the uterine horn, spleen and large tracts of the omentum attached to the intestine (Figure **3A** **, lower panel**). Moderate activity was seen in the brain, bone and perigonadal and inguinal fat deposits.

In the brain of both sexes strong AR activity was detected in specific regions of the brain upon coronal dissection, including the cerebral cortex, thalamus and pituitary gland (Figure **3B& C**).

### Reporter activity is maintained in cultured primary cells

Primary ARE-Luc mouse cells were cultured from various tissues, and their steroid receptor and luciferase reporter gene expression were analysed further. Pre-adipocyte cells expressing AR, GR, ERα/β and PR (as analysed by quantitative PCR Figure **S3A**), were starved for 48hours and treated with various steroid hormones. Luciferase expression was induced by androgen treatment, specifically mibolerone, testosterone and to a lesser extent with androstenedione (Figure **S3B**). Neither dexamethasone nor progesterone induced luciferase activity in these cells, although GR and PR expression predominate. Primary cells derived from the salivary glands, uterus and the liver also produced varying amounts of luciferase activity in culture when treated with mibolerone (Figure **S3C**).

### Androgen induced luciferase activity is inhibited by anti-androgen bicalutamide

**Figure 4:**
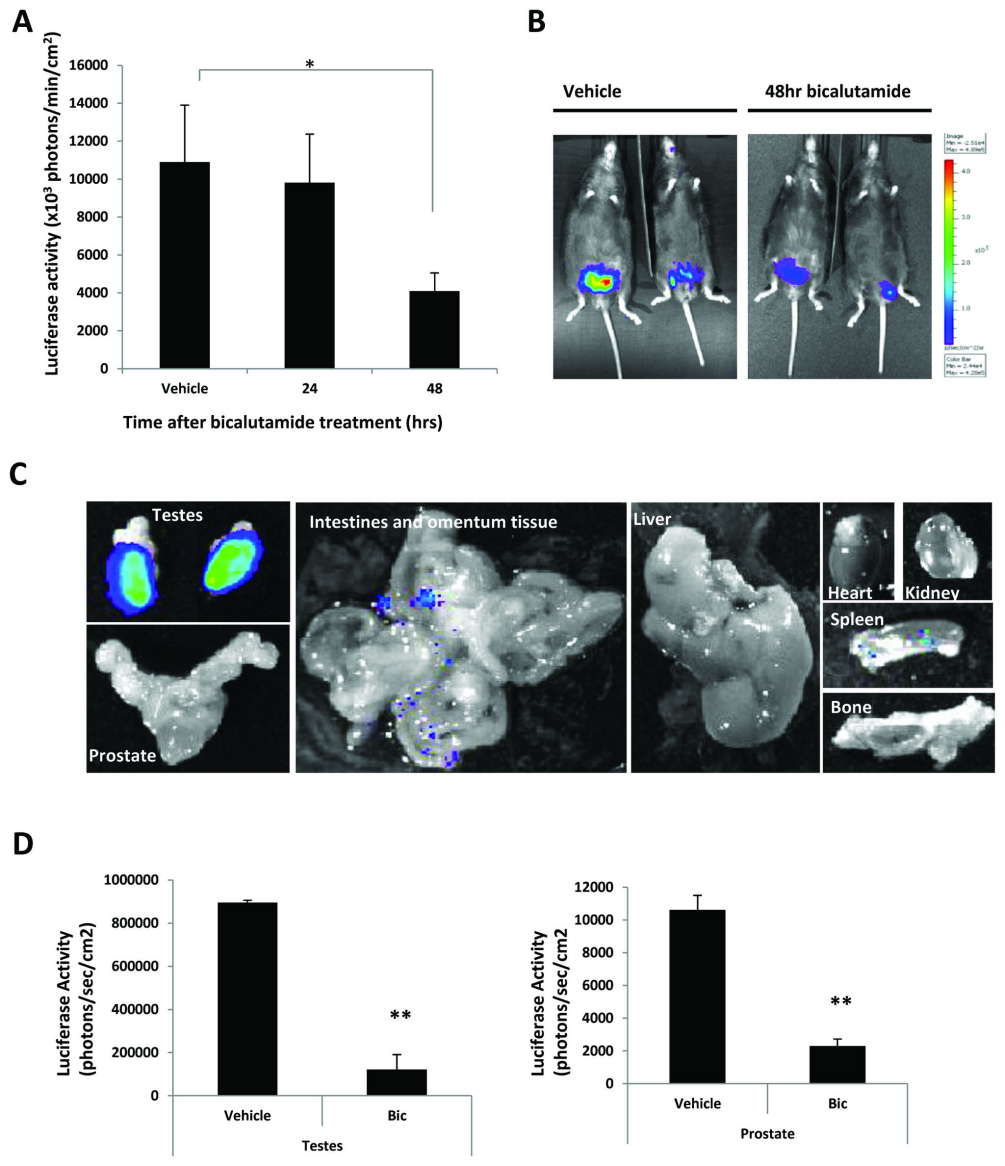
Bicalutamide treatment reduces androgen receptor activity in male mice. Bioluminescent imaging of male mice treated with bicalutamide (50mg/kg) for 24 and 48 hours. (A), Graph indicates measured bioluminescence signal from the gonadal region at the indicated timepoints; error bars represent the standard error from three mice in each group. (B), representative bioluminescent image of vehicle and bicalutamide treated mice at 48hours. Figure represents a greyscale photograph overlaid with a pseudocolour representation of bioluminescence; scale represents photons/sec/cm^2^. (C), panel showing *ex vivo* imaging of the organs from bicalutamide treated mice after 48hours (intestines image not to scale). (D), Bioluminescence signal from the testes and the prostate taken ex vivo, from male mice treated for 48hours with vehicle or bicalutamide. Error bars represent the standard error from three mice. **P<0.01, *P<0.05 (t-test analysis).

To further demonstrate that luciferase expression is mediated by the AR, intact male ARE-Luc mice (aged 6-12 weeks) were treated with 50mg/kg/day of the antiandrogen bicalutamide, in DMSO: propylene glycol (50:50), for 48hrs. Mice were imaged at 24 and 48 hours and compared to vehicle treated controls. Bicalutamide treatment reduced luciferase expression in the gonadal region of the ARE-Luc mice at 24hrs and significantly so at 48hrs (Figure **4A& B**). *Ex vivo* organ imaging revealed that bioluminescence was visibly reduced in all tissues after 48hr bicalutamide treatment (Figure **4C** **, compared to** Figure **3A**). Bioluminescence in intestine, heart and bone was barely detectable, while the prostate and testes showed, respectively, approximately 80 and 90% reduction (Figure **4D**).

**Figure 5:**
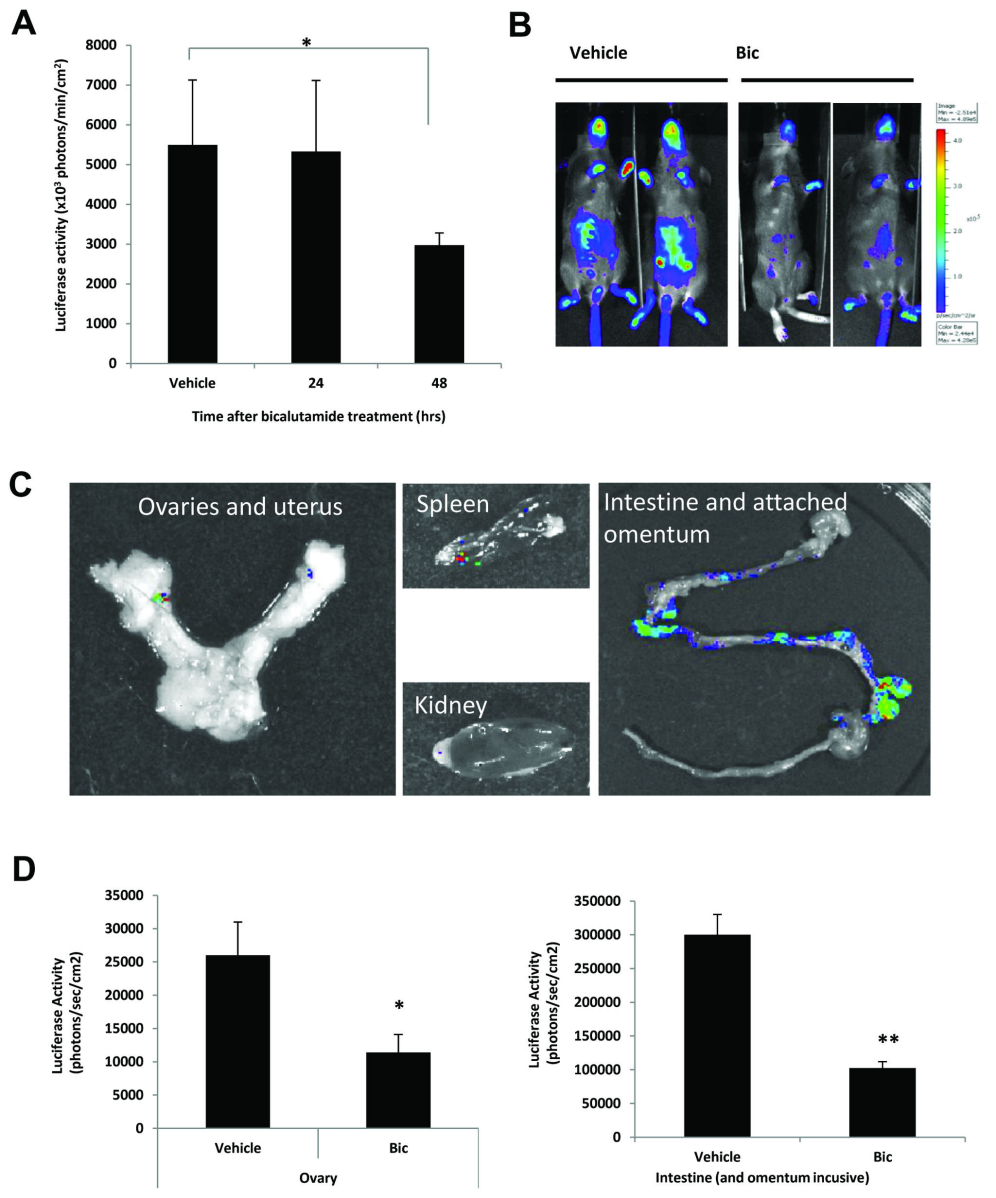
Bicalutamide treatment reduces androgen receptor activity in female mice. (A), Bioluminescent imaging of female mice treated with bicalutamide (50mg/kg) for 24 and 48 hours. Graph indicates measured bioluminescence signal from the abdominal region at the indicated timepoints, error bars represent the standard error from three mice. (B), representative bioluminescent image of vehicle and bicalutamide treated mice at 48hours. Figure represents a greyscale photograph overlaid with a pseudocolour representation of bioluminescence; scale represents photons/sec/cm^2^. (C), panel showing *ex vivo* imaging of the organs from bicalutamide treated mice after 48hours (intestines image not to scale). (D), Bioluminescence signal from the ovaries and the intestines taken ex vivo, from female mice treated for 48hours with vehicle or bicalutamide. Error bars represent the standard error from three mice, **P<0.01, *P<0.05 (t-test analysis).

Females showed a similar reduction of luciferase activity when treated with 50mg/kg bicalutamide. Overall, whole body luciferase signal was strongly reduced after 48 hours, and specifically the combined abdominal and mammary gland signal was reduced approximately 70% (Figure **5A& B**). *Ex vivo* imaging of the organs confirmed that bioluminescence had dropped significantly in the intestine, ovary, and uterus (Figure **5C& D** **, and compare to** Figure **3A**).

### Luciferase expression in tissues from ARE-Luc mice correlates with AR expression

**Figure 6:**
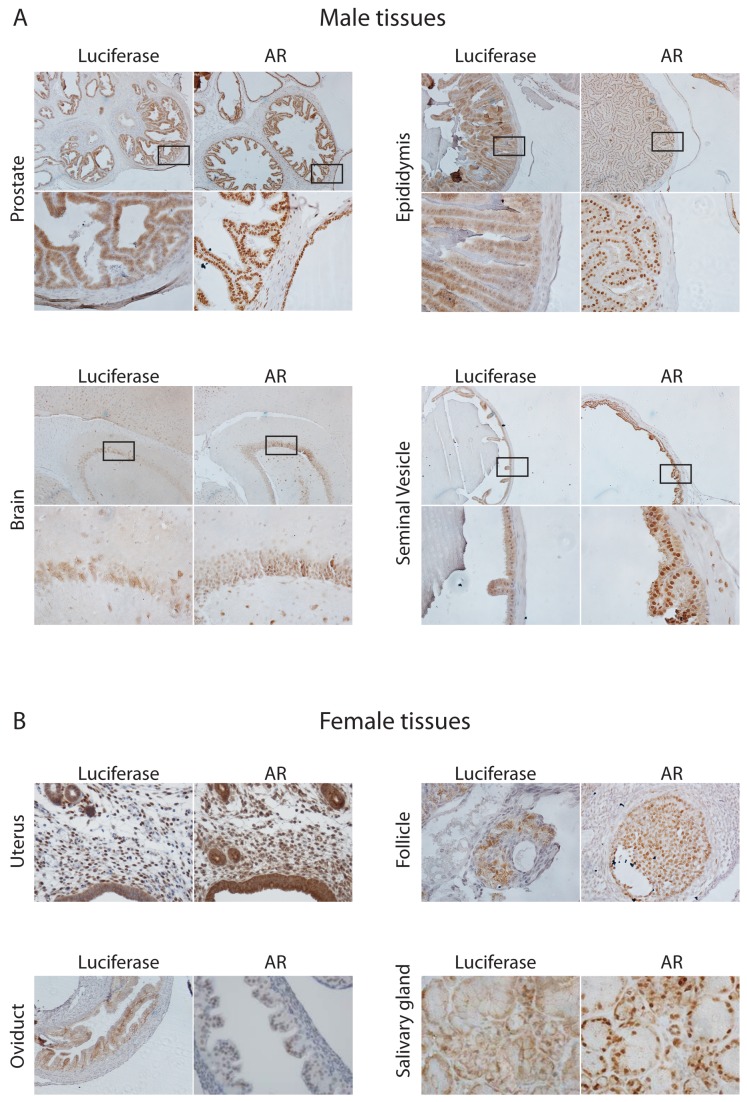
Analysis of AR and luciferase expression within a sample of mouse tissues. (**A**), Immunohistochemical co-localisation staining for AR (nuclear and cytoplasmic_ and luciferase (cytoplasmic) on consecutive formalin-fixed tissue sections taken from the ARE-Luc male mice. Upper panel represents image at 10x magnification, with box inset and lower panel showing 40x magnification. (B), Immunohistochemical staining for AR in a variety of female mouse tissues.

To confirm that luciferase is expressed in the AR-positive cell types, a selection of the strongly AR expressing tissues were also immunostained for luciferase expression. Adjacent sections of tissues from ARE-Luc mice were stained with antibodies against AR and luciferase. AR immunoreactivity was detected in the nuclei of prostate, epididymal and seminal vesicle epithelial cells in the male, as well as in distinct cell layers of the cerebral cortex (Figure **6A**). In the female, AR immunoreactivity was detected in the nuclei of ovarian follicles (granulosa cells), oviduct epithelial cells, salivary glands, and uterine epithelial cells (Figure **6B**). Cytoplasmic staining for luciferase was seen in the same cell types and cell layers as nuclear AR staining (Figure **6A& B**).

**Figure 7:**
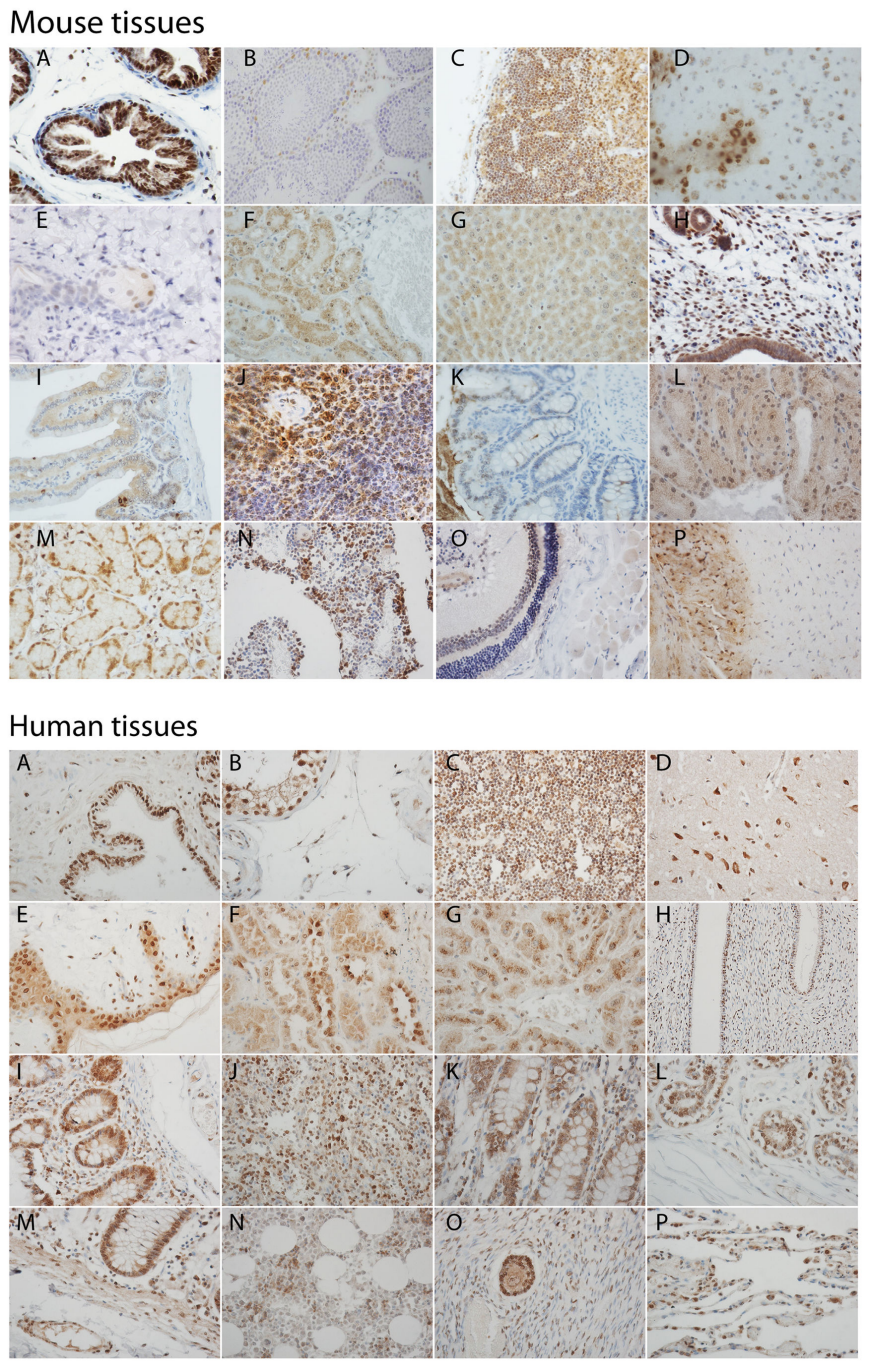
Upper panel - Mouse tissues: A, prostate; B, testis; C, thymus; D, brain; E, skin and hair follicle; F, kidney; G, liver; H, uterus; I, intestine; J, spleen; K, colon; L, lacrimal gland; M, salivary gland; N, bone marrow; O, eye; P, heart. Lower panel - Human tissue: A, prostate; B, testis; C, thymus; D, brain; E, skin; F, kidney; G, liver; H, uterus; I, intestine; J, spleen; K, colon; L, breast; M, intestine and omentum; N, bone marrow; O, ovary and follicle; P, lung.

Tissues expressing AR correlated with those demonstrating luciferase activity (Figures **2B** and **3**), e.g. brain, testes, prostate, ovaries, uterus and mammary glands. Certain tissues did not show a close correlation e.g. AR levels were high in the salivary glands, but luciferase activity was relatively low (see Figures **2B** and **6**). Tissues such as the omentum and the eye (and associated lacrimal glands) showed modest AR staining (Figure **7**) but relatively robust luciferase activity (Figure **2B**). Presently we do not know the mechanism behind such differences; they may be due to local androgen levels, and/or local co-activator/co-repressor ratios within tissues. However, importantly, no luciferase activity was detected in tissues without AR expression.

### AR expression patterns are similar in mouse and human tissues

Formalin-fixed wild-type mouse tissue sections as well as a multiple organ human normal tissue microarray were stained for AR (Figure **7A& B**). Mouse tissues positive for AR expression showed very similar staining patterns to the respective human tissue (Table **1**). We did not take into account any gross morphological differences in tissue – especially relevant for the brain and prostate.

**Table 1:** Summary of the immunohistochemical analysis of AR in mouse tissue sections, and human normal tissue microarray.

**Tissue**	**Staining pattern**	**Intensity mouse**	**Intensity human**
Adipose tissue	Weak staining, nuclear	+	+
Bladder wall	Occasional nuclei in stromal cells	+	NA
Bone marrow	Strong nuclear, 50% of cells	+++	++
Brain	Strong nuclear, multiple neuronal cell types, highly regional	++	++
Caecum	Weak nuclear	+	+
Colon	Mixed cytoplasmic and nuclear staining.	+	+
Epididymis	Nuclear and cytoplasmic in luminal epithelial cells	+++	NA
Eye	Weak nuclear on inner retinal layer	+	NA
Fallopian tube & oviduct	Strong nuclear stain	+++	NA
Heart	Cytoplasmic staining, area specific nuclear staining	+	NA
Kidney	Weak cytoplasmic granular, nucleoli	+	++
Liver	Weak cytoplasmic	+	+
Lung	Occasional positive nucleus	+	+
Muscle (leg)	Very weak nuclear stain	+	+
Omentum	Nuclear stain in some cell layers and tubules	+	+
Optic nerve-associated glands	Granular cytoplasmic, weak nuclear	+	NA
Ovary	Strong nuclear stain in granulosa cells	+++	++
Prostate	Nuclear in luminal epithelial cells	++++	++++
Salivary gland	Strong nuclear and cytoplasmic	+++	NA
Seminal Vesicle	Nuclear and cytoplasmic	+++	++
Skin	Nuclear staining in specific epidermal layers and hair follicles	++	++
Small intestine	Weak / cytoplasmic	+	+
Spleen	Nuclear, also cytoplasmic in female	+++	+++
Stomach	Cytoplasmic and nuclear in stomach wall	+	++
Testis	Nuclear in Sertoli and Leydig cells	++	++
Thymus	Strong nuclear	++	++
Uterus	Strong nuclear stain in some layers	++	++

## Discussion

### The ARE-Luc mouse: a model for AR activity

AR drives transcriptional networks in varied tissue types and is responsible for the development, maintenance and function of several tissues in the body. However, relatively few androgen-regulated genes have been well characterised, most driving functions of the prostate e.g. those encoding kallikrein enzymes (*PSA* and *KLK2*) and, in rodents, probasin (PB). There is a need for models to study steroid receptor activity in physiological settings and the first mouse model allowing visualisation of endogenous steroid receptor activity was developed for the ER by Maggi and co-workers [27,28]. To develop a similar model for AR, it was necessary to circumvent the issue that, unlike ER, AR binds a common response element shared by several other steroid receptors. Our ARE-Luc model utilizes an ARE that has been extensively tested *in vitro* to minimise cross talk with other steroid receptors, with specific emphasis on optimising the ARE type to exclude GR binding and activation. In a previous model, the AR activity indicator (ARAI) mouse, steroid receptor crosstalk is avoided by expressing an engineered AR, in which the native DNA-binding domain (DBD) is replaced by the Gal4-DBD, and a Gal4-responsive luciferase reporter is used [29]. This study showed lower AR activity than expected, possibly due to lack of appropriate interaction domains required for maximal AR activity [30,31]. The DBD is instrumental in driving the androgen-specific genome response, as evidenced by altered genomic responses in a model with the AR DBD replaced by that of the GR (the SPARKI mouse) [32], and suggests a non-native DBD could well influence the downstream response to AR activation and representative AR responses will depend upon the presence of intact AR.

The single *SC*1.2 ARE was weaker than *PSA* regulatory AREs, but unlike these it did not show significant response to GR [22,26]. Further, the exclusion of adjacent sequences that contain binding sites for other transcription factors eliminates the potential for tissue specific expression – the opposite strategy was used in previous transgenic models that were designed to give prostate specific expression [25,33,34]. To avoid positional effects due to random integration as seen for other models, e.g. that using the ARE from Sex-limited protein (Slp) gene enhancer [35], we used a knock-in strategy into a non-silenced genomic locus (*Hprt*). The use of a highly active luciferase (*luc2P*) coupled to degradation signals ensured sufficient activity for rapid and robust detection, with opportunity for rapid flux in response to changes in hormone levels. We acknowledge that a plethora of transcription factors and co-activator proteins may act in concert with the AR to fine-tune activation but no one model could reproduce this level of complexity, and therefore our model reports and assays the activity of transcriptionally active AR.

### AR activity in male and female ARE-Luc mice

Luciferase activity was seen only in those tissues expressing AR, and the reporter was silent in tissues lacking AR, indicating no constitutive activation or silencing due to positional integration effects although it is impossible to completely rule out “false negative” results due to the unlikely scenario of an as yet undetected tissue specificity of the reporter minimised by use of an artificial promoter containing a minimal ARE. Importantly, a strong reduction in luciferase signal was seen when AR was inhibited by bicalutamide – even though androgens were still circulating in the intact animal – demonstrating that the luciferase activity in these mice is indeed mediated by the AR. Although the signal in the majority of luciferase-expressing organs became almost undetectable in both sexes following anti-androgen treatment, some residual signal was seen in the most strongly positive organs e.g. the testes. This is likely due to incomplete inhibition by the competitive anti-androgen bicalutamide, as testes are known to have the highest tissue testosterone concentrations [36]. A more complete control to demonstrate the dependence on AR activity would be to cross the mice with an AR-deficient mouse such as the Tfm or ARKO model [8,37]; however, this is problematic due to the X-chromosomal location of both the *AR* gene and the *Hprt* locus into which the luciferase reporter is inserted.

*Ex vivo* tissue examination showed exquisitely specific localisation of luciferase activity, for example in the gonads and brain (indicating the ability of luciferin substrate to cross the blood brain barrier) in both sexes, which correlated well with AR protein expression within tissues (In turn, sites of AR expression in mouse correlated with expression in human tissues). Within the brain, in both sexes, AR activity was highly localised to specific regions, detailed study of which will be the focus of future research. Several reports have suggested that within the brain testosterone activity is mediated by ER after aromatisation of testosterone, especially during foetal development. Certain areas of the brain are more susceptible to aromatisation and these areas adhere well to the theory, e.g. the sexually dimorphic nucleus of the preoptic area and the anteroventral periventricular nucleus [38]. However, AR has been well documented to be present in the brains of both males and females in mammals, with highly complex functions; further, aromatisation does not account for all the sexual differentiation in brain morphology and in animal behaviour [39–41]. Complete androgen insensitivity syndrome (due to lack of AR function) presents with a female habitus and physiology, including the developmental brain pattern - in spite of internalised testes that secrete normal levels of testosterone [42]. This indicates a requirement for AR signalling in brain masculinisation. Further, human males with aromatase mutations present as male with a masculinised brain pattern, indicating a limited role for ER [43]. We observed high AR protein levels in certain regions of the brain via immunohistochemistry, concomitant with luciferase staining and strong luciferase activity in *ex vivo* tissue. Our model supports the hypothesis that aromatisation is not the only mechanism of androgen activity in the brain, and that AR is transcriptionally active within certain brain regions. It is not possible to state, at this stage, which androgen is mediating the effect, but future crosses of ARE-Luc mice with a 5α-reductase knockout mouse should clarify whether testosterone (aromatisable) or DHT (non-aromatisable) is responsible.

Another regions showing high luciferase activity was the gastric tract, notably the surrounding intestinal omentum. The omentum represents a complex tissue that has multiple roles including fat storage [44,45] and immunological responses [46,47] – both roles with a strong androgen stimulus (e.g. spleen, bone marrow, thymus and adipose tissue all show robust AR and luciferase activity). There are contradictory reports of AR expression in the colon. Although we saw low to moderate staining for AR and luciferase in the small intestine and colonic surfaces, including the villi, proteinatlas.org and other reports indicated no staining for AR in these tissues [48,49]. However, others have found AR expression in several compartments of the mammalian intestine [50] and have indicated a role for AR in the intestine in modulating ion channels, glucose and calcium uptake [51–54]. Interestingly, androgens also affect smooth muscle activity in contractile peristalsis [55],. Our staining is consistent with this since we see AR positivity in surrounding muscle as well as the intestine itself, hence the luciferase signal observed could be from a combination of these. The role of androgens in intestinal/omentum activity requires further analysis.

This study shows that the AR is indeed very active outside its well-defined role in reproductive tissues. We have observed AR expression and activity in a variety of tissues in which the role of steroid hormones are poorly understood. The retina and choroid cells of the eye showed modest AR expression and luciferase activity, as did the lacrimal glands – tissues known to express sex steroid receptors [56,57]. Strong AR expression and activity was seen in the immunological system, including the bone marrow, spleen and thymus. AR expression in the spleen is somewhat controversial, for instance immunohistochemical staining for AR in all mouse tissues by Takeda et al. did not not show reactivity [58]. However, more recent studies have shown modest levels of AR expression and an important role for testosterone in the functioning of the immune system, for instance splenic B cells express AR in mice, as do leukocytes from both spleen and thymus in rats [59,60]. The AR activity seen by luciferase imaging is thus likely to be due to the immune cell contents of the spleen and indeed AR immunoreactivity is not uniform across the spleen tissue (Figure 7). Interesting, sex differences have been observed in a number of models of autoimmune diseases, reflecting the well-known gender bias in humans. Androgens tend to have a suppressive effect upon the immune system, and castration (or lack of transcriptionally active functional AR) increases lymphocyte numbers in both thymus and spleen [37,61–63]. Tissues such as the heart and the brain showed highly regional activity and expression both within the tissue itself and exhibited differences between the sexes. The model shows clearly that androgens have a plethora of effects in males as expected, but also demonstrates the importance of this signalling pathway in females.

In summary, we report a highly specific model for imaging endogenous AR activity in mice that overcomes previous limitations including crosstalk with other receptors, random integration, non-endogenous AR and tissue-specific expression. Our model shows that androgens have a wide range of target tissues, both in males and females, outside the most widely-studied reproductive tract. The data shown here represent a snapshot of the AR activity within adult mice (6-10 weeks), thus exclude any dramatic changes predicted during development and puberty, as well as effects of hormonal fluctuations during the female estrous cycle and less dramatic changes during aging. As well as such developmental studies, future uses of this model include evaluation and detection androgenic xenobiotics and selective androgen receptor modulators (SARMs) with the aim of producing tissue-selective antagonistic or agonistic effects in diseases such as breast and prostate cancer, polycystic ovarian syndrome and hyper- and hypogonadism [64].

## Materials and Methods

### Ethics statement

All mouse procedures were performed in accordance with the UK Animals (Scientific Procedures) Act 1986 under Home Office license.

### Reporter construction

A 168 bp fragment containing the minimal thymidine kinase (tk) promoter from the herpes simplex virus was cloned into the Sac1/Xho1 site of pGL4.18 (Promega) to generate p-*tk*-Luc. DNA oligomers (EurofinsMWG, Germany) coding for the SC1.2 ARE sequence (GGCTCTttcAGTTCT) were ligated into the Sac1/Xba1 site of p-*tk*-Luc to generate p-*tk*-Luc-ARE (Figure **1A**). This construct was tested for androgen specificity and inducibility, see Dart et al. 2009 and supplemental data therein.

### Transgenic mice

The *S*.C. 1.2 ARE-Luc knock-in (ARE-Luc) mouse was generated in collaboration with GenOway (Lyon, France). The p-*tk*-Luc-ARE was cloned into the CBE1-HR Hypoxanthine phosphoribosyltransferase gene (*Hprt*) conditional targeting vector, containing the *Hprt* promoter and exon 1. This gene-targeting construct was introduced into male mouse embryonic stem (E14Tg2a ES) cells of the 129P2/OlaHsd strain in which the 35kb of the *Hprt* gene, encompassing the 5’ UTR up to intron 2, is deleted. The targeting construct then integrated into the X-chromosome by homologous recombination (Figure **S1A**). ES cells were selected using HAT medium (Hypoxanthine, Aminopterin and Thymidine). Homologous recombination was confirmed by Southern blotting (Figure **S1B**). The modified ES cells were inserted into C57BL/6J blastocysts, which were then implanted into the uterus of OF1 pseudo-pregnant female mice to generate chimeric offspring. The chimeric males were crossed with C57BL/6J strain wild-type females to generate F1 mice (Figure **S1C**), from which the heterozygous females were used for breeding with wild-type C57BL/6J males to generate hemizygous males and heterozygous females (Figure **S1D**). Homozygous females were produced at the following generation by back-crossing (Figure **S1E**). All offspring were genotyped by PCR of genomic DNA extracted from ear notching.

### Luciferase assay

Tissue was pulverized by grinding in liquid nitrogen and then completely homogenised, using a microfuge pestle, in reporter lysis buffer with protease inhibitors (Promega). Lysate (20µl) was mixed with luciferin substrate (20µl) and light emission measured using the Steadylite luciferase assay kit (PerkinElmer, U.K.) in a Topcount luminometer (Packard Instrument Co, USA). Light expression was then normalised to protein content as measured by Bradford Assay.

### Luciferase imaging

Anaesthetized mice (3% isofluorane with O_2_ carrier, Abbott Animal Health UK) were injected *i.p.* or *s.c.* with D-luciferin (Caliper Life Sciences Ltd, Runcorn, UK) at 150 mg/kg, 10 min before imaging. Light emission from luciferase was detected by the IVIS Imaging System 100 series (Xenogen Corporation), and overlaid as a pseudocolour image with reference scale, upon a greyscale optical image.

For *ex vivo* imaging, mice were sacrificed 10 min after luciferin injection, and immediately dissected. Target organ was rinsed briefly in PBS and placed under the bioluminescent camera.

### Histology and immunohistochemistry

Standard protocols were carried out as described [65]. Antibodies used were: AR (N-20 Santa Cruz) @1:300, and Luciferase (Promega). The Vectastain avidin–biotin complex (Vector Labs, Peterborough, U.K.) was used for detection, using diaminobenzidine chromogenic substrate. Negative controls lacking primary antibody were also carried out. Digital images were captured using E1000 microscope (Nikon, Kingston upon Thames, UK) and Eclipse Net image analysis software.

### Additional Information

The ARE-luc mouse line will be made available for non-profit research use: for details please contact the corresponding author Dr C. L. Bevan.

## Supporting Information

Figure S1 **Southern blot validation of the 5’ and 3’ homologous recombination event.** A, Schematic representation of the wild type, deleted and the recombined Hprt allele with the relevant restriction sites for the Southern blot analysis shown. Black lines represent the homology arms. The Southern blot strategy for the detection of the 5’ and 3’ targeting events is indicated by arrows. B and C, Southern blot analysis of genomic DNA of the tested ES clones and the wild-type C57Bl/6 Hprt allele probed with 5’ probe D and 3’ probe A.D, validation of the F1 generation, and E, validation of the N2 generation.
(TIF)

Figure S2 **Luciferase kinetics depends on route of injection.** Comparison of the time kinetics of bioluminescent signal from ARE-Luc mice injected with 150mg/kg luciferin substrate via either the *i.p.* or *s.c.* route.
(TIF)

Figure S3 **Analysis of luciferase activity and expression in mouse ARE-Luc primary cells in culture.** **A**, Relative mRNA expression of steroid hormone receptors in gonadal pre-adipocytes. **B**, Luciferase activity in gonadal adipose cells grown under conditions of hormone starvation for 72 hours and treated with 10nM hormone for 24 hours. **C**, Luciferase expression in various primary cell types, hormone-starved for 72 hours and treated with mibolerone (10nM) or equivalent volume vehicle (Eth) for 24 hours.
(TIF)

Methods S1 Further details are provided for Cell culture, Primary Cell Culture, Genomic DNA extraction and PCR, RNA extraction, RT-PCR and Southern Blotting in the Supporting Information for this paper.
(DOCX)
