# Supplementary figures and images for: Visualising Androgen Receptor Activity in Male and Female Mice
Source: PLoS One. 2013 Aug 7;8(8):e71694. doi: 10.1371/journal.pone.0071694 (PMC3737126; doi:10.1371/journal.pone.0071694)

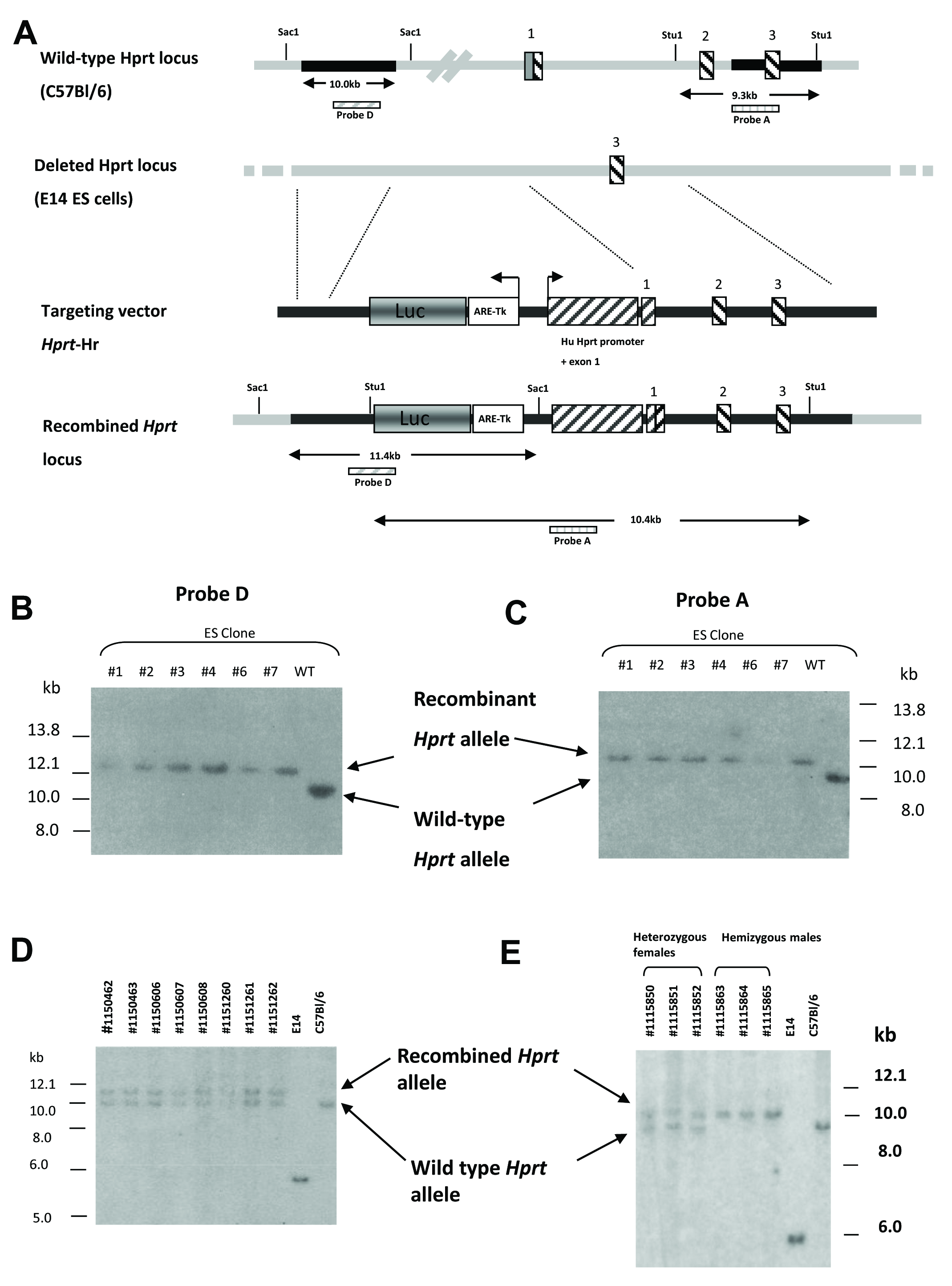

Supplement: Figure S1 — Southern blot validation of the 5’ and 3’ homologous recombination event. A, Schematic representation of the wild type, deleted and the recombined Hprt allele with the relevant restriction sites for the Southern blot analysis shown. Black lines represent the homology arms. The Southern blot strategy for the detection of the 5’ and 3’ targeting events is indicated by arrows. B and C, Southern blot analysis of genomic DNA of the tested ES clones and the wild-type C57Bl/6 Hprt allele probed with 5’ probe D and 3’ probe A.D, validation of the F1 generation, and E, validation of the N2 generation. (TIF) [file pone.0071694.s001.tif]

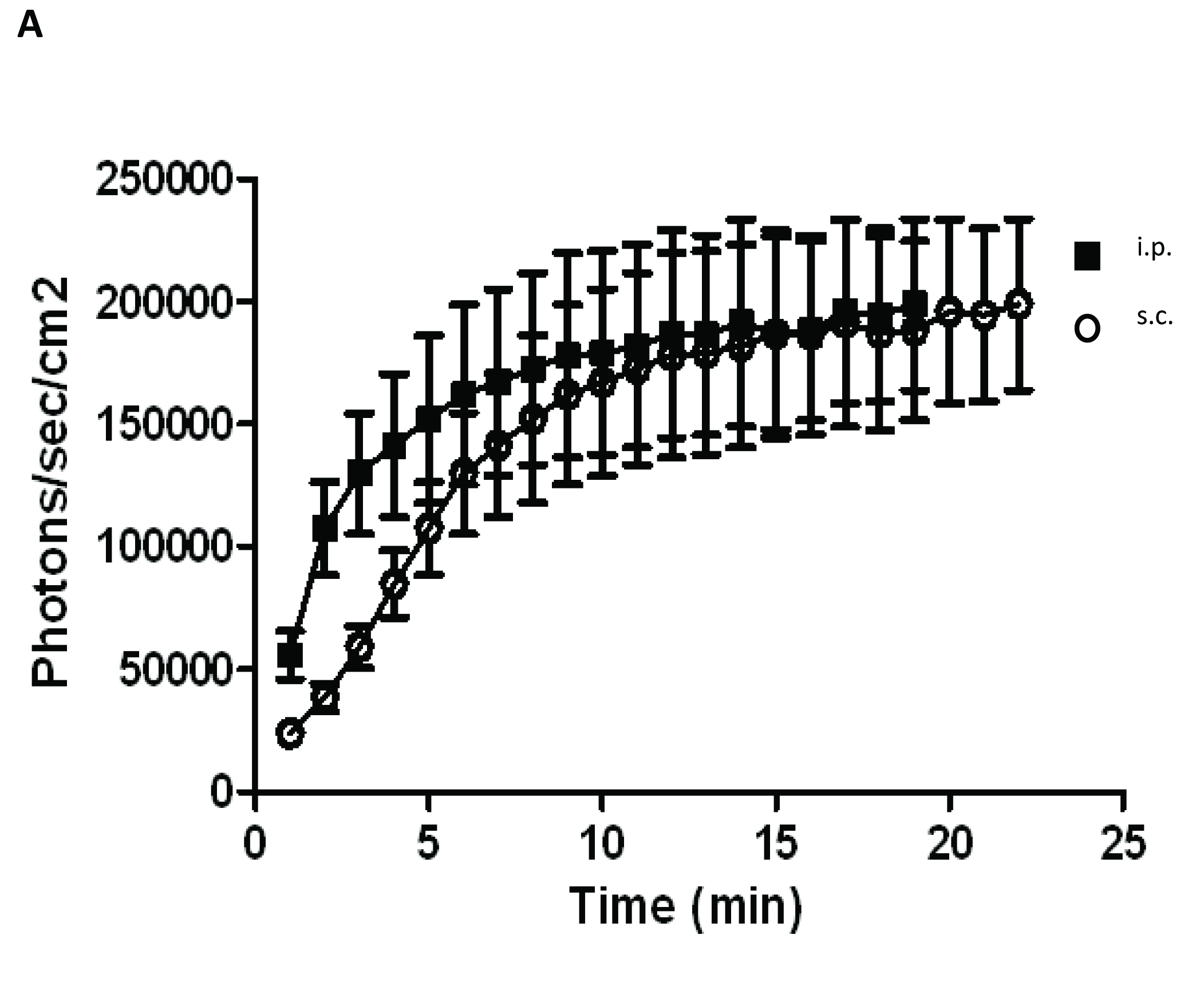

Supplement: Figure S2 — Luciferase kinetics depends on route of injection. Comparison of the time kinetics of bioluminescent signal from ARE-Luc mice injected with 150mg/kg luciferin substrate via either the i.p. or s.c. route. (TIF) [file pone.0071694.s002.tif]

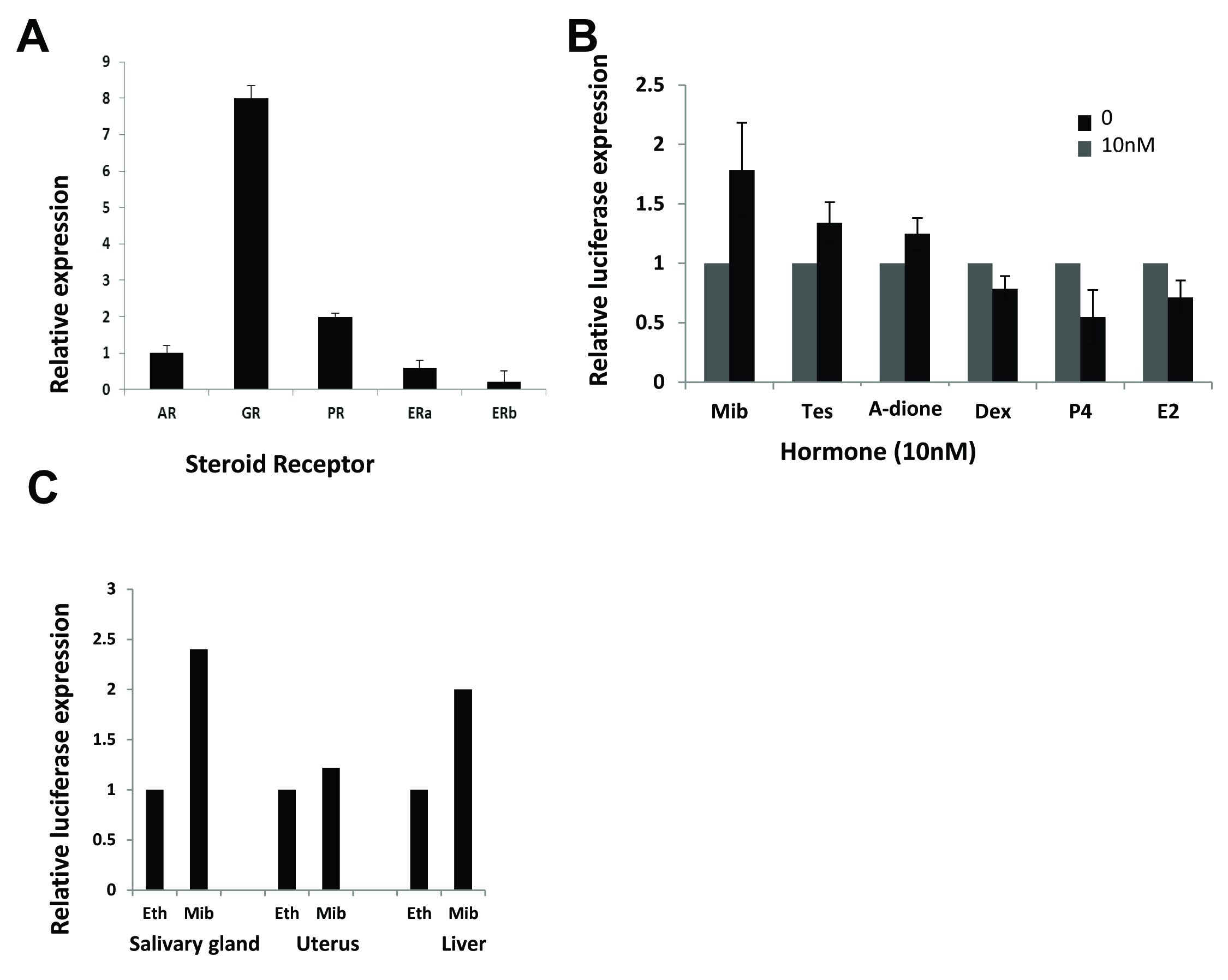

Supplement: Figure S3 — Analysis of luciferase activity and expression in mouse ARE-Luc primary cells in culture. A, Relative mRNA expression of steroid hormone receptors in gonadal pre-adipocytes. B, Luciferase activity in gonadal adipose cells grown under conditions of hormone starvation for 72 hours and treated with 10nM hormone for 24 hours. C, Luciferase expression in various primary cell types, hormone-starved for 72 hours and treated with mibolerone (10nM) or equivalent volume vehicle (Eth) for 24 hours. (TIF) [file pone.0071694.s003.tif]
