# Supplementary material for: Visualising Androgen Receptor Activity in Male and Female Mice
Source: PLoS One. 2013 Aug 7;8(8):e71694. doi: 10.1371/journal.pone.0071694 (PMC3737126; doi:10.1371/journal.pone.0071694)
Supplement: Methods S1 — Further details are provided for Cell culture, Primary Cell Culture, Genomic DNA extraction and PCR, RNA extraction, RT-PCR and Southern Blotting in the Supporting Information for this paper. (DOCX) [file pone.0071694.s004.docx]

**Dart et al.**

**Supplemental methods**

**Cell culture**

All cells were maintained at 37^o^C, 5% CO_2_, in media containing 2 mM L-glutamine, 100 units/ml penicillin and 100 mg/ml streptomycin (Sigma). LNCaP/Luc and Du145 cells were maintained in RPMI medium (Sigma) supplemented with 10% foetal bovine serum (Clontech) with 12 μg/ml blasticidin (Invitrogen), and G418 (500 μg/ml). MCF-7 and COS-1 cells were maintained in DMEM medium (Sigma) supplemented with 10% foetal bovine serum (First Link UK, Ltd, Brierley Hill, UK), with additional blasticidin (12μg/ml), and G418 (500μg/ml) for the MCF-7/Luc cells. Twenty-four hours before exposure to androgen, medium was replaced with ‘starvation medium’ consisting of phenol red-free RPMI or DMEM medium as appropriate supplemented with 5% charcoal-stripped foetal bovine serum (First Link UK). GnWAT cells were maintained in F12:DMEM (50:50) with 10% foetal bovine serum and 2 mM L-glutamine, 100 units/ml penicillin and 100 mg/ml streptomycin.

**Primary cell culture**

Tissue was cut finely into small squares (approximately 0.5 mm3) using scalpel and forceps. Approx. 0.3 - 0.5ml of 0.5% collagenase, and 1 x trypsin EDTA (Sigma) was added to the pieces and they were digested at 37° C with gentle agitation for 1-4 hours. 10ml of media (F12:DMEM 50%:50%, with 10% FCS) was added to the digested tissue pieces and the cell suspension was taken and transferred to a T25 flask at 37° C, 5% CO_2_. The flask was left undisturbed for 2-3 days, then examined under a microscope for cell adhesion. Cells were washed several times to remove any debris and red blood cells. Cells were then passaged every week, or when required.

**Genomic DNA extraction and PCR**

Tissue was ground in lysis buffer (100mM Tris-HCl pH 8.5, 5mM EDTA, 0.2% SDS, 200mM NaCl), using a microfuge pestle, and were digested with proteinase K (200μg/ml) for 4-6hours at 56°C, with gentle agitation. Samples were then centrifuged at 13,000 x g and the supernatant was mixed with 100% isopropanol to precipitate genomic DNA. The DNA pellet was then washed with 70% ethanol and resuspended in dH_2_O.

**RNA extraction**

Total RNA samples were prepared using commercial kits (Qiashredder and RNeasy kits from Qiagen) according to the manufacturer’s instructions. After extraction, the RNA was treated with DNaseI (Qiagen) to eliminate genomic DNA contamination, and then eluted. The RNA was quantified by measuring the absorbance at 260 nm. 1μg of total RNA was converted to cDNA using the SuperScript First-Strand Synthesis system (Invitrogen), utilising Oligo(dT)_12-18_ primers for full length cDNA or random hexamers for RT-PCR detection.

**RT-PCR**

RT-PCR for prostate-specific antigen (PSA) and L19 control was carried out as described [38]. *Luciferase* (200bp fragment) was detected using the primer pairs 5’-gctcagcaaggaggtaggtg-3’ and 5’-tcttaccggtgtccaagtcc-3’ with 24 cycles of 94 ^o^C for 30 s, 54 ^o^C for 30 s and 70 ^o^C for 1 min. Mouse *androgen receptor* was detected using the primer pairs 5’-tagggctgggaagggtctac-3’ and 5’-tgcttaagcctgggaaagtg-3’ with 22–24 cycles of 94 ^o^C for 30 s, 54 ^o^C for 30 s and 70 ^o^C for 1 min. The mouse wild type *Hprt* allele was detected using the primer pair 5’-tgtccttagaaaacacatatccagggtttagg-3’ and 5’-cggtgggacatttgagttgcttgc-3’, with 35 cycles of 94 ^o^C for 30 s, 65 ^o^C for 30 s and 70 ^o^C for 5 min. The recombined Hprt-luc-ARE allele was detected using the primer pair 5’-acgtcagtagtcataggaactgcggtcg-3’ and 5’-cggtgggacatttgagttgcttgc-3’ with 35 cycles of 94 ^o^C for 30 s, 65 ^o^C for 30 s and 70 ^o^C for 1 min.

TaqMan quantitative PCR assays for each gene target were performed in triplicate on cDNA samples in 96-well optical plates on an ABI Prism 7700 Sequence Detection system (PE Applied Biosystems). For each 20μl TaqMan reaction, 2μl cDNA was mixed with 7μl PCR-grade water, 10μl 2xTaqMan Universal PCR Master Mix (PE Applied Biosystems) and 1μl Taqman assay probe. PCR parameters were 50 ^o^C for 2 min, 95 ^o^C for 10 min, 40 cycles of 95 ^o^C for 15 s and 60 ^o^C for 1 min. Data were recorded using Sequence Detector Software (SDS version 2.3; PE Applied Biosystems). From each amplification plot, a threshold cycle (Ct) value was calculated, representing the PCR cycle number at which the fluorescence was detectable, based on the variability of base line data in the first 15 cycles. PSA levels were normalized to glyceraldehyde- 3-phosphate dehydrogenase (GAPDH) levels.

**Southern Blotting**

Genomic DNA was digested with the desired restriction endonucleases overnight, and then run on a 1% agarose gel, alongside a digested pMAR.ARE vector control. Agarose gels were then denatured in 1.5M NaCl, 0.5M NaOH for 30minutes, before being neutralized in 1M Tris pH 7.5. DNA was transferred to positively charged nylon membrane by capillary transfer overnight, in 10xSSC buffer. DNA was fixed to the membrane by baking at 80^o^C for 30minutes.

Membranes were then pre-hybridized using PerfectHyb Plus buffer (Sigma) and were then probed for *Luciferase* using the digoxygenin-labelled probe oligo 5’-cccagtgtcttaccggtgtccaagtccaccaccttagcctc-3’ (MWG-Eurofin, Germany) overnight at 68^o^C. Membranes were washed in 0.1xSSC, 0.5% SDS for 3x20min and then with TBS. Membranes were then blocked with 5% BSA, and then digoxygenin was detected using alkaline phosphatase coupled anti-digoxygenin Fab fragment (Roche). Enzymatic activity was then detected using either chemoluminescent CDP-Star substrate (Roche) using photographic film, or developed by colourimetrically using blue BCIP/NBT substrate (Vector).

The internal probe D (507bp) was generated by PCR using the following primers; 5’-ctcatttgttcaagaccagcctgtgctacc-3’ and 5’-attaataacttcttttcagacaagtagagattgc-3’, and was then radiolabelled. Probe A was generated by PCR using the following primers 5’tctataggactgaaagacttgctcg-3’ and 5’-acttacacagtagctcttcagtctg-3’ and radiolabelled.
